# Sin Nombre Virus Infection in Field Workers, Colorado, USA

**DOI:** 10.3201/eid1602.090735

**Published:** 2010-02

**Authors:** Fernando Torres-Pérez, Linda Wilson, Sharon K. Collinge, Heath Harmon, Chris Ray, Rafael A. Medina, Brian Hjelle

**Affiliations:** University of New Mexico, Albuquerque, New Mexico, USA (F. Torres-Pérez, B. Hjelle); Boulder County Public Health, Boulder, Colorado, USA (L. Wilson, H. Harmon); University of Colorado, Boulder (S.K. Collinge, C. Ray); The Mount Sinai School of Medicine, New York, New York, USA (R.A. Medina); 1 These authors contributed equally to this article.

**Keywords:** Sin Nombre virus, viruses, rodents, vector-borne infections, hantavirus, field workers, Colorado, dispatch

## Abstract

We report 2 cases of Sin Nombre virus (SNV) infection in field workers, possibly contracted through rodent bites. Screening for antibodies to SNV in rodents trapped in 2 seasons showed that 9.77% were seropositive. Quantitative real-time PCR showed that 2 of 79 deer mice had detectable titers of SNV RNA.

Hantaviruses are rodent-borne viruses that in the Americas have been implicated as the causative agents of hantavirus cardiopulmonary syndrome (HCPS) (*1*). In North America, Sin Nombre virus (SNV) is responsible for most cases of HCPS, and the deer mouse (*Peromyscus maniculatus*) is its main reservoir. Since the first reported outbreak of the disease in 1993 in the southwestern United States, rodent serologic surveys have confirmed that SNV is present through most of the range where deer mice exist, including Colorado (*2*,*3*). We report 2 cases of SNV infection in field workers in Colorado, who were infected with the virus despite protection with a powered air-purifying respirator. We also performed a serologic survey of wild rodents in the presumptive areas of exposure and quantitative real-time PCR analyses of blood samples from deer mice identified as seropositive.

## The Study

In this study of human infections by SNV in Boulder County, Colorado, we identified 2 patients who had trapped rodents for ecologic studies. On June 14, 2005, a 24-year-old man was admitted to Boulder Community Hospital with fatigue, headache, fever, and thrombocytopenia (70,000 platelets/μL) but without cardiorespiratory compromise. A strip immunoblot assay identified immunoglobulin (Ig) M and IgG against SNV N and Gn proteins. On July 6, 2005, a 22-year-old IgM- and IgG-seropositive woman was admitted to Boulder Community Hospital with fever and dyspnea; she subsequently experienced bilateral lung infiltrates and thrombocytopenia (116,000 platelets/μL). She required oxygen supplementation but recovered almost completely by July 11. She reported performing fieldwork in the same period as did patient 1 but with no overlap among the sites (the distances between sampling sites where the 2 field workers most probably contracted their infections ranged from 6.4 to 9.8 km). Both patients engaged in field activities involving manipulating traps and rodents in areas where deer mice were seropositive for SNV (Table).

**Table:** Mammal species, abundance, seropositivity, and Sin Nombre virus RNA quantification during May–June and August–September 2005, Boulder, Broomfield, and Jefferson counties, Colorado, USA*

Species (common name)	No. animals			No. seropositive			Antibody prevalence, %		SNV titers
	May–Jun	Aug–Sep		May–Jun	Aug–Sep		May–Jun	Aug–Sep	
*Chaetodipus hispidus* (hispid pocket mouse)	23	58		0	2		0	3.44	
*Cynomys ludovicianus* (black-tailed prairie dog)	171	33		0	0		0	0	
*Microtus ochrogaster* (prairie vole)	26	11		0	1		0	9.1	
*Microtus pennsylvanicus* (meadow vole)	10	1		2	0		20	0	
*Mus musculus* (house mouse)	1	2		0	0		0	0	
*Neotoma mexicana* (Mexican woodrat)	3	0		0	0		0	0	
*Peromyscus maniculatus* (deer mouse)	711	780		105	82		14.77	10.5	Animal 1050, 520.8 copies/mL; Animal 2404, 87.45 copies/mL
*Reithrodontomys megalotis* (western harvest mouse)	8	21		1	4		12.5	19.0	
*Spermophilus tridecemlineatus* (thirteen-lined ground squirrel)	3	4		0	0		0	0	
*Sylvilagus audubonii* (Audubon's cottontail)	1	1		0	0		0	0	

*SNV, Sin Nombre virus.

Along with the 2 patients, another 15 field workers were surveyed to assess possible exposures to SNV. Ninety-five questions were asked involving, among others, contacts with rodents and use of personal protective measures and equipment. Most (83.3%, 14/15) reported previous experience with rodents in the field; all workers were required to wear nitrile gloves and use a powered air-purifying respirator when handling animals. No differences in risk exposure to contract hantavirus were evident between infected and noninfected persons. Six persons reported having been bitten >1 times by rodents, including both case-patients. Patient 2 was bitten twice, with 1 bite resulting in bleeding despite the worker’s use of nitrile gloves. Patient 1 reported being bitten by a vole (*Microtus* sp.) on June 2, and patient 2 was bitten by 2 deer mice on June 14. Their wounds were treated by immediate cleaning and bandaging. Patient 2 also applied an antimicrobial ointment before bandaging her bleeding wound.

We hypothesized that the workers might have been exposed to a subset of rodents with unusually high titers of SNV. Therefore, we resampled sites in Boulder and Jefferson counties in Colorado (where the field workers were infected) during August–September 2005. A total of 44 sites were sampled during both trapping periods by using live traps (H.B. Sherman Traps, Tallahassee, FL, USA) in grids for 4 consecutive nights. Prairie dogs (*Cynomys* *ludovicianus*) were also trapped at a subset of these study sites during June–July 2005 with live traps (Tomahawk Live Traps, Tomahawk, WI, USA). A total of 1,868 animals from 10 mammalian species were captured during both trapping periods (Table).

We screened blood samples by strip immunoblot assay for antibodies against SNV N protein (*4*). Four rodent species yielded positive samples from 197 blood samples. Deer mice showed the highest abundance of seropositive samples, although harvest mice (Reithrodontomys *megalotis*), which carry El Moro Canyon virus, had higher seroprevalence. Two (2.5%) of 81 hispid pocket mice (*Chaetodipus hispidus*) were also positive but are unlikely to play an epidemiologic role. Small mammal capture frequencies varied during the 2 sampling periods; seroprevalence for pocket mice, prairie voles, and harvest mice increased, and that for meadow voles (*M. pennsylanicus*) decreased. Seroprevalence among deer mice was higher during May–June (when the field workers contracted their infections) than in August–September.

We performed TaqMan (Applied Biosystems, Foster City, CA, USA) quantitative real-time PCR on a subset of 79 (of 187) samples from deer mice that had detectable antibodies to SNV N antigen. The samples selected for PCR analysis were those for which the volume of blood was deemed sufficient (>25 μL) to carry out a satisfactory RNA extraction. We chose 25 μL as the minimal amount for detecting SNV small segment RNA by nested reverse transcription–PCR on the basis of a spiking experiment in which 5 μL of lung homogenate from an infected deer mouse had been added to 20 μL of blood from an uninfected deer mouse, resulting in a positive finding. The equivalents of 10-μL aliquots of total blood RNA (RNeasy Mini Kit; QIAGEN, Valencia, CA, USA) were subjected to quantitative real-time PCR with primers, probes, and PCR conditions as described (*5*). We detected only low levels of SNV in the blood of 2 of the 79 seropositive deer mice tested (Table). This low number of samples with detectable SNV RNA (2.53%) is congruent with previous findings reporting undetectable levels of SNV RNA in blood using quantitative real-time PCR (*6*).

## Conclusions

The primary mode of hantavirus transmission to humans is through rodent excreta and secretions through the aerosol route (*7*). Although indoor exposure in poorly ventilated buildings has been reported as a major factor for contraction of HCPS, our survey supports the possibility that the 2 patients contracted SNV outdoors and that, in at least in 1 case, a rodent bite was the proximate vehicle for transmission of SNV to the field worker. This route of transmission is uncommon with only few examples reported (*8*–*10*). The fact that patient 1 was bitten by a vole and not by a deer mouse does not necessarily exclude transmission of SNV by that route. Voles are not known to transmit SNV, but there have been repeated instances of vole-associated hantaviruses being carried by sigmodontine rodents (*11*). Thus, sigmodontine-borne hantaviruses might also replicate productively in voles. Although the power of this survey is limited by small sample size, we believe that our finding are potentially useful and suggest that increased attention be devoted toward avoiding rodent bites among the handlers of wild rodents in regions where hantaviruses occur. Although both workers sustained rodent bites, 1 by a known SNV carrier and 1 by another rodent species, one should remain open-minded about the actual route of infection, which might still be through an airborne route rather than through bites in either case.

Our results suggest that detecting SNV RNA of sufficient magnitude (>80 copies/mL) to score as positive in TaqMan assays might be uncommon in the natural reservoir. Therefore, high loads of SNV RNA might not be a major factor in virus transmission in the wild. Alternatively, SNV might cause only a brief RNA viremia in wild deer mice (*12*), and possibly the small number of real-time PCR–positive deer mice represents those animals that underwent recent seroconversion. This phenomenon has also been observed with other rodent borne-hantaviruses (*13*). Alternatively, or in addition, the small number of mice found to have quantifiable viral RNA in this study might be a consequence of physiologic events (such as viral recrudescence) (*14*), which result in intermittent detection of viral RNA in blood, a phenomenon that might be shared by other agents of hemorrhagic fevers (*15*).
